# Genomic Variation Among and Within Six *Juglans* Species

**DOI:** 10.1534/g3.118.200030

**Published:** 2018-05-23

**Authors:** Kristian A. Stevens, Keith Woeste, Sandeep Chakraborty, Marc W. Crepeau, Charles A. Leslie, Pedro J. Martínez-García, Daniela Puiu, Jeanne Romero-Severson, Mark Coggeshall, Abhaya M. Dandekar, Daniel Kluepfel, David B. Neale, Steven L. Salzberg, Charles H. Langley

**Affiliations:** * Department of Evolution and Ecology; ‡ Department of Plant Sciences University of California, Davis, CA 95616; † USDA Forest Service Hardwood Tree Improvement and Regeneration Center, Department of Forestry and Natural Resources, Purdue University, West Lafayette, IN; § Center for Computational Biology, McKusick-Nathans Institute of Genetic Medicine; ‡‡ Departments of Biomedical Engineering, Computer Science, and Biostatistics, Johns Hopkins University, Baltimore, MD; ** Department of Biological Sciences, University of Notre Dame, IN; †† USDA Agricultural Research Station, Davis, CA

**Keywords:** *Juglans*, walnut, reference genomes, genomic variation, polyphenol oxidase

## Abstract

Genomic analysis in *Juglans* (walnuts) is expected to transform the breeding and agricultural production of both nuts and lumber. To that end, we report here the determination of reference sequences for six additional relatives of *Juglans regia*: *Juglans sigillata* (also from section *Dioscaryon*), *Juglans nigra*, *Juglans microcarpa*, *Juglans hindsii* (from section *Rhysocaryon*), *Juglans cathayensis* (from section *Cardiocaryon*), and the closely related *Pterocarya stenoptera*. While these are ‘draft’ genomes, ranging in size between 640Mbp and 990Mbp, their contiguities and accuracies can support powerful annotations of genomic variation that are often the foundation of new avenues of research and breeding. We annotated nucleotide divergence and synteny by creating complete pairwise alignments of each reference genome to the remaining six. In addition, we have re-sequenced a sample of accessions from four *Juglans* species (including *regia*). The variation discovered in these surveys comprises a critical resource for experimentation and breeding, as well as a solid complementary annotation. To demonstrate the potential of these resources the structural and sequence variation in and around the polyphenol oxidase loci, *PPO1* and *PPO2* were investigated. As reported for other seed crops variation in this gene is implicated in the domestication of walnuts. The apparently *Juglandaceae* specific PPO1 duplicate shows accelerated divergence and an excess of amino acid replacement on the lineage leading to accessions of the domesticated nut crop species, *Juglans regia* and *sigillata*.

The family Juglandaceae contains approximately seven genera and 59 species distributed worldwide across temperate and tropical regions (Manning 1978). Many walnut species are valued for human use as timber or nut trees, particularly species within *Juglans*, *Carya*, and *Pterocarya*, (Hu *et al.* 2016; Grauke *et al.* 2016) and the hard masts produced by many species are also an important sources of nutrition for wildlife (Perkey and Wilkins 2001; MacGowan 2003; Hui-jin and Bo-gen 2005). Because of their economic importance, genetic resources for members of the Juglandaceae have been developed and used worldwide for breeding, conservation, and forest management (Woeste and Michler 2011; Ebrahimi *et al.* 2016; Stone *et al.* 2009; McGranahan and Leslie 2012; Grauke *et al.* 2016). Two important examples are the development and application of microsatellites (Pollegioni *et al.* 2014; Wang *et al.* 2016; Gunn *et al.* 2010) and the availability of chloroplast sequences to define phylogenetic relationships (Aradhya *et al.* 2006; Hu *et al.* 2016) and evolutionary history (Smith and Doyle 1995; Laricchia *et al.* 2015).

As genomic resources become available, the application of genomics to problems of breeding and forest management is expanding rapidly (Neale *et al.* 2013; Holliday *et al.* 2017). The recent publication of a draft reference genome for *J. regia* (Martínez‐García *et al.* 2016), a physical map (Luo *et al.* 2015), as well as numerous transcriptomes (Chakraborty *et al.* 2016; Dang *et al.* 2016; Qiu *et al.* 2016) will accelerate the use of genomics for the Juglandaceae. As genomic data and tools become more widely available, applications are emerging in many areas including comparative genomics (Neale and Kremer 2011; Krutovsky *et al.* 2004; Lee *et al.* 2003), phylogenomics (Stölting *et al.* 2013), functional genomics (Neale and Ingvarsson 2008; Evans *et al.* 2014; Plomion *et al.* 2016, Du and Groover 2010; Liu *et al.* 2015), the microbiomes (Cordier *et al.* 2012), epigenomics (Bräutigam *et al.* 2013; Gao *et al.* 2014), and of course, breeding (Resende *et al.* 2012).

Here, we add to the existing *J. regia* reference genome of the widely planted Chandler cultivar (Martínez‐García *et al.* 2016) by describing draft nuclear reference genomes for *five* additional members of *Juglans* (*J. nigra*, *J. hindsii*, *J. microcarpa*, *J. sigillata*, *J. cathayensis*) as well as the Chinese wingnut (*Pterocarya stenoptera*), another member of the walnut subfamily *Juglandoideae*. *J. nigra*, *J. hindsii* and *J. microcarpa* are native to the United States and are members of the New World section of *Juglans* (Rhysocaryon). *J. sigillata*, *J. cathayensis*, and *P*. *stenoptera* are native to China. *J. regia* and *J*. *sigillata* are the only members of section Dioscaryon/Juglans. *J. sigillata* may be a sub-species or ecotype of *J*. *regia* (Wang *et al.* 2008). Chinese walnut (*J. cathayensis*) is a member of section Cardiocaryon that grows in central and southern China and is weakly differentiated (if at all) from *J. mandshurica* which has a more northerly distribution (Brach and Song 2006; Aradhya *et al.* 2007; Bai *et al.* 2016).

Eastern black walnut (*J. nigra*) is a common tree native to the mesic hardwood forests of the Eastern United States (Williams 1990; Reid *et al.* 2004; Shifley 2004; Michler *et al.* 2007). It is valued for its timber (Beineke 1983; Settle *et al.* 2015) and its nuts, which are processed for both industrial and food products (Hammons 1998). Genetic resources for *J. nigra* are second only to *J. regia* among the *Juglans* in terms of number and types (Woeste and Michler 2011). The northern California black walnut (*J. hindsii*), also called Hinds black walnut, is thought to be native to a small region of northern California but to have spread via cultivation across a much wider area of California and Oregon (McGranahan and Leslie 1991). Hybrids between Hinds black walnut and *J. regia* are known as Paradox (Matheron and Mircetich 1985; Baumgartner *et al.* 2013). Paradox are widely deployed as rootstocks for commercial orchards of *J. regia*. *Juglans microcarpa*, also called Texas black walnut, grows in isolated, favorable riparian habitats in the arid plains of the United States and northern Mexico. Although it is more a shrub than a tree, it has value in rootstock breeding as well (McGranahan and Leslie 2009). *J. sigillata* is a medium-sized tree found on mountain slopes in southern China and in Tibet (Brach and Song 2006). *Pterocarya stenoptera* or Chinese wingnut is a vigorously growing tree that can reach 30 m in height (Brach and Song 2006). Itis frequently cultivated as a shade tree (its winged nut is small and inedible). Although it is resistant to several important pests and diseases of walnut and can be used as a rootstock, its hybrids with *J. regia* are non-viable (McGranahan *et al.* 1986).

As a demonstration of the utility of these genomes, the origins and evolutionary relationship of polyphenol oxidases (PPO) genes in *Juglans* is investigated. PPO genes are copper-binding enzymes that oxidize ortho-diphenols to ortho-quinones in the pathway involved in the browning reactions that occur after tissue damage (Jiang 2000). Reduction of PPO function has been implicated in domestication associated grain colors of three species of Asian rice (Yu *et al.* 2009), barley (Taketa *et al.* 2010) and foxtail millet (Inoue, *et al.* 2015). PPO genes are also implicated in the plant defense response (Thipyapong *et al.* 2004; Li and Steffens 2002; Richter *et al.* 2012). The number of PPO genes varies from zero in *Arabidopsis* (Tran *et al.* 2012) to 19 in *Salvia miltiorrhiza*, an important ingredient in traditional Chinese medicine (Li *et al.* 2017). Previously, genomic resources established that *J. regia* actually has two PPO genes, *JrPPO1* was the first to be found and characterized (Escobar *et al.* 2008), while the complete genome revealed the presence of another gene, expressed at much lower levels (*JrPPO2*) and in a narrow range of tissue types (Martínez-García *et al.* 2016). These genomes are used to more broadly investigate the origins and evolutionary relationship of PPO genes in *Juglans*. The recently solved structure of *JrPPO1* provides a unique perspective to evaluate how evolutionary forces may have influenced protein function.

A detailed and thorough comparative analysis of the genomes of these species is beyond the scope of this paper, but to foster the application of genomics in *Juglans* research and breeding, we report here the sequencing and assembly of the genomes of five additional *Juglans* species. Second, we provide accessible *pairwise* alignments of these genomes annotating synteny and between-species divergence. Finally, an important adjunct to the genome structures and divergences revealed in these resources are surveys of the within species genomic polymorphisms for four species. Beyond the obvious value of detected SNPs as a resource for the development of genotyping tools, genomic variants can be annotated with respect to their potential phenotypic consequences (Cingolani *et al.* 2012; McLaren *et al.* 2016), thus serving as a readily accessible source of candidates in functional genomic analyses and gene-oriented breeding and biotechnology. We report the resequencing of samples of independent accessions of two *Juglans* species with scion breeding programs, *J. regia* and *J. nigra*, as well as two species involved in rootstock development, *J. hindsii* and *J. microcarpa*. Species differences in the overall levels of genomic polymorphism are documented. Polymorphism is used in conjunction with divergence to infer recent selection possibly associated with domestication. Finally, we demonstrate how these genomic resources can be visualized in support of gene-oriented analyses by employing the widely used and well-supported JBrowse software (Skinner *et al.* 2009).

## Material And Methods

### DNA Extraction

Nuclei were isolated from adult leaves of each species as previously described (Zimin *et al.*, 2014). Nuclei were lysed by adding N-laurylsarcosine to a final concentration of 1% (w/v) and incubating for 15 min at room temperature. 5M NaCl and 10% (w/v) cetyltrimethyl ammonium bromide (CTAB) were added to final concentrations of 0.7M and 1% (w/v) respectively and the mixture was incubated at 60° for 30 min. DNA was then extracted twice with an equal volume of chloroform:isoamyl alcohol (24:1), precipitated with 2/3 volume of 100% isopropanol, and re-suspended in TE buffer.

### Library construction and Sequencing

Two types of libraries were prepared from the resulting DNA as follows (using enzymes and buffers from New England Biolabs unless otherwise indicated):

#### Paired end Libraries:

DNA (5 µg) was fragmented by sonication in a Diagenode Bioruptor NGS instrument (high power setting, 9 cycles of 15 sec on, 90 sec off). Fragments were end-repaired in a 100 µl reaction in 1X T4 ligase buffer containing 0.4 mM (each) final concentration of dNTPs, 15 U T4 DNA polymerase, 50 U T4 polynucleotide kinase and 5 U DNA polymerase I large (Klenow) fragment. End-repaired fragments were A-tailed in a 50 µl reaction in 1X NEBuffer 2 containing 0.2 mM final concentration of dATP, and 15 U of Klenow fragment (exo-). Paired-end adapter was prepared by heating an equimolar mixture of two HPLC-purified oligos (5′-ACACTCTTTCCCTACACGACGCTCTTCCGATOT and 5′-PHO- GATCGGAAGAGCACACGTCT where 5′-PHO indicates 5′ phosphorylation and O indicates C with a phosphothioate linkage to the next base on the 3′ side) in a tube immersed in ∼500 ml of boiling water and then leaving the tube immersed while the water cooled slowly to room temperature. Annealed adapters were ligated to A-tailed fragments in a 50 µl reaction in 1X Quick Ligation buffer containing 3 µM final concentration of paired-end adapter, and 5 µl of Quick T4 ligase. Adapter-ligation product was size-selected on a 2% agarose gel in 1X TAE run until the bromophenol blue band had migrated approximately 9 cm. Two ∼1 mm-thick slices were then cut from the gel under blue light transillumination at a position approximately adjacent to the 500 bp ladder band and DNA was extracted from each slice using the MinElute Gel Extraction kit (Qiagen). Concentration of the recovered DNA was estimated using an Agilent Bioanalyzer 2100, and 10 ng of DNA from each slice was used as template in a 50 µl PCR reaction in 1X KAPA HiFi HotStart ReadyMix (KAPA Biosystems) containing PAGE-purified barcoded primers at 0.5 µM each (forward primer 5′-AATGATACGGCGACCACCGAGATCTACACTCTTTCCCTACACGACGCTCTTCCGATOT and reverse primer either 5′-CAAGCAGAAGACGGCATACGAGATGTAGCCGTGACTGGAGTTCAGACGTGTGCTCTTCCGATOT or 5′-CAAGCAGAAGACGGCATACGAGATTACAAGGTGACTGGAGTTCAGACGTGTGCTCTTCCGATOT where O indicates C with a phosphothioate linkage to the next base on the 3′ side). Cycling parameters were 5 min at 95° followed by 10 cycles of 20 sec at 98°, 30 sec at 65°, and 30 sec at 72°, followed by a final 5 min extension step at 72°. DNA purifications following all reactions listed above were performed using PCRClean DX beads from Aline Biosciences according to the manufacturer’s instructions.

For *J. nigra* and *J. sigillata* an additional short fragment library per species was made using the Illumina TruSeq DNA PCR-Free Sample Prep Kit following the manufacturer’s instructions for 550 bp target insert size.

#### Mate pair Libraries:

DNA (15 µg) was treated with 5 ul of PreCR Repair Mix (New England Biolabs) in a 450 ul reaction in 1X ThermoPol buffer containing 0.1 mM (each) final concentration of dNTPs and 0.5mM final concentration of NAD+. DNA was then purified by one extraction with phenol/chloroform/isoamyl alcohol (25:24:1) and one extraction with chloroform followed by ethanol precipitation. 4 µg of PreCR-repaired DNA was used as input for the Nextera Mate Pair Sample Preparation kit (Illumina) following the manufacturer’s “gel plus” protocol. Size selection was performed with a BioRad FIGE Mapper using a buffer re-circulating pump and the following conditions: 1X TAE buffer; 16 hr run at room temperature; 4.1 V/cm forward and 2.7 V/cm reverse field strength, both with linear ramping from 0.1 sec initial to 0.8 sec final switch time. Gel slices were cut from the gel adjacent to ladder bands at ∼3kb, ∼6 kb and ∼10 kb. Circular ligation products were fragmented by sonication in a Diagenode Bioruptor NGS instrument (high power setting, 5 cycles of 15 sec on, 90 sec off). Fifteen cycles of enrichment amplification were performed.

Completed libraries were pooled as necessary and sequenced in paired-end Rapid Run mode on a HiSeq 2500 (Illumina). Read lengths were 151 bp forward read and 151 bp reverse read. Sequencing results for each library are given in Table S1.

### Assemblies

Genomes were assembled following the method used in (Martínez-García *et al.* 2016). Scaffolds originating from uncollapsed heterozygous sequence were expected. We partially addressed this issue by identifying and completely removing nested redundant scaffolds as follows: we aligned all scaffolds shorter than 50 Kb to each other using bwa (Li and Durbin 2009) and MUMmer (Kurtz *et al.* 2004), and used the show-coords program within MUMmer to identify scaffolds that were completely contained by and nearly identical to other, longer scaffolds.

### Pacbio sequencing and re-assembly of *J. regia*

Light coverage of longer reads (PacBio) was obtained in an effort to improve the existing *J. regia* genome assembly. DNA was extracted from adult leaves from the same tree used for the original *J. regia* genome (Martínez-García *et al.* 2016) and subsequently converted into a sequencing library using the method previously described (Zimin *et al.* 2017). In total, 814,584 PacBio sequence reads were obtained totaling 6 billion bp (∼10 fold coverage of the genome). These were combined with previous Illumina data and assembled using the MaSuRCA hybrid assembly method described in Zimin *et al.* (2017). The resulting unannotated assembly is included here as an additional resource and to confirm results inferred from micro-synteny to the original assembly.

### k-mer Analysis

For each species, 31-mer histograms were computed using the software jellyfish (Marçais and Kingsford 2011) on the paired end Illumina reads. The command jellyfish ‘count (-m 31 -s 1G–bf-size 200G)’ was used to generate each database, and jellyfish histo was used to compute each histogram. The program jellyfish query (-s) was used to obtain the depth of 31-mers for specific sequences. Custom scripts were used to perform the set operations required for the PPO depth analysis. Genome sizes were estimated from 31-mer histograms using the method described in (Sork *et al.* 2016).

### Pairwise genome alignment

Pairwise genome alignment was conducted with the nucmer nucleotide alignment software component of the mummer v4.0 software package. Each genome participated as a query and as a reference in the alignments. Genome alignment was conducted using the command ‘nucmer–prefix = ref_qry ref.fasta qry.fasta’ recommended for aligning draft genomes to draft genomes. Alignments were then filtered using the nucmer command ‘delta-filter’ to select the best weighted set of non-overlapping alignments to the query that cover the reference sequence. Alignments were then processed using the ‘show-coords’ command with a minimum reference sequence length of 1000 bp. Genome wide coverages of the alignments were calculated as the number of aligned query bases over the total number of bases in the reference genome over 1000bp. Genome wide divergences were calculated as the number of mismatches, from the nucmer percent identity, over the number of aligned query bases.

### Core Gene Annotation and PPO Analysis

To annotate a “core” set of genes expected to be present in each assembly, version 2.5 of the Core Eukaryotic Genes Mapping Approach (CEGMA) (Parra *et al.* 2007) and version 2.0 of Benchmarking Universal Single-Copy Orthologs (BUSCO) (Simão *et al.* 2015) were run on each genome separately. CEGMA was run using default parameters and BUSCO was run in ‘geno’ mode using the ‘embryophyta_odb9’ profile.

The PPO genes annotated using GMAP (Wu and Watanabe 2005) to align the two available *J. regia PPO1* and *PPO2* sequences (Martínez‐García *et al.* 2016) to each of the additional genome assemblies analyzed in this paper. The inferred nucleotide sequences for each copy were obtained from the alignments. A few genomes contained more than one copy of a gene, in each case, a copy confirmed by synteny (on the same scaffold) was considered the ortholog.

### Phylogenetic tree construction

To construct a phylogenetic tree from pairwise genome alignments, estimates of pairwise divergence were calculated from the average genome alignment divergence values and corrected using the method of Jukes and Cantor (1969). The unrooted tree was then constructed using the Neighbor-Joining method (Saitou and Nei 1987) as implemented by the ‘neighbor’ program in the ‘Phylip’ package (Felsenstein 1986). The outgroup *P. stenoptera* was excluded from this method due to the low coverage observed in the pairwise alignments.

Genome wide phylogenetic trees were also constructed using the filtered and curated multiple alignments of single copy BUSCO genes present in all seven species. For each BUSCO gene annotated as single copy in all seven species, multiple alignments of both protein and nucleotide sequences were constructed with the software MUSCLE (Edgar 2004). Gap columns were subsequently filtered for phylogenetic reconstruction, and if an alignment consisted of 50% or higher gap columns, it was completely filtered. Filtered alignments were concatenated, resulting in a total input dataset of 373,615 sites. The phylogenetic tree was inferred by Maximum Likelihood using PhyML (Guindon and Gascuel 2003). To test for phylogeny 100 bootstrap replicates were performed.

Divergence times for all branching points in the topology were calculated using the Maximum Likelihood method based on the model of Tamura and Nei (1993). A chronogram was subsequently estimated using the method of Tamura *et al.* (2012) using the *Juglans* crown group ancestor as the calibration point. The 95% confidence intervals shown were computed using the method described in Tamura *et al.* (2012).

A gene tree for the PPO family was constructed from the orthologous nucleotide sequences annotated in each genome. From the nucleotide sequences a multiple sequence alignment was created using MUSCLE (Edgar 2004). A maximum likelihood phylogenetic tree was constructed using PhyML (Guindon and Gascuel 2003) using 100 bootstrap replicates. Lineage specific K_a_ and K_s_ values were then estimated using this tree using the method of (Zhang *et al.* 2006).

### Single Nucleotide Polymorphisms

Reads were aligned to the genome using bwa mem (Version 0.7.13; Li and Durbin 2009). Aligned reads were subsequently converted into bam format using samtools view (Version 1.3; Li *et al.* 2009). The bam-formatted files were sorted using samtools sort. A multi-sample vcf file was then produced for SNP calling using samtools mpileup. SNPs were called using the multi-sample allele calling algorithm of bcftools call (Version 1.2; Li 2011) with reads from each sample distinguished. SNP filtering was performed using bcftools view. To classify a subset of diploid sites, histograms of aligned read depth were used to inform the minimum and maximum coverage cutoffs for a SNP in each species (Table S2). Nucleotide diversity π (Nei and Li 1979) was estimated from the intermediate vcf files. To account for variation in coverage, nucleotide diversity was calculated as a site weighted average for each of the different coverage classes.

### Data availability

The genomic resources described here are available at NCBI under bioproject PRJNA445704 and through hardwoodgenomics.org. Re-sequencing data are available at the NCBI SRA under study SRP149991. Supplemental material available at Figshare: https://doi.org/10.25387/g3.6328697.

## Results

### Estimation of genome size and relative heterozygosity

For each target genome, we deeply sequenced paired end and mate-pair libraries (Table 1; Table S1). Every base of each genome was represented an average of 100 times in the whole genome shotgun Illumina sequence data from paired end libraries. For the very long insert mate-pair libraries used for linking, we looked at the *physical* coverage based on the input DNA molecule, which is much longer than the reads. For each genome, we sequenced libraries in long insert size ranges of about 3000, 5000, and 10000 bp, targeting at least 100-fold physical coverage in all cases (Table S1).

**Table 1:** Selected targets and the basic statistics for the unassembled genomes. The genome sequences assembled in this study all have 2n = 32 chromosomes. The Illumina sequence reads and the corresponding genome sizes estimates from the 31-mer analysis of the paired end reads are given. Qualitative levels of heterozygosity, 1 = highest 7 = lowest, are based on quantitatively ranking the 31-mer distributions by relative proportion of the two peaks

Taxonomy Properties	*Juglans. hindsii*	*Juglans nigra*	*Juglans cathayensis*	*Juglans microcarpa*	*Juglans sigillata*	*Juglans regia*	*Pterocarya stenoptera*
**Chrom. num.**							
(2n)	32	32	32	32	32	32	32
**Plant**							
name	‘Rawlins’	‘Sparrow’	‘Wild Walnut’	’83-129’		‘Chandler’	’83-13’
accession	DJUG105		DJUG11.03	DJUG29.11	DJUG951.04	64-172	DPTE1.09
source	NCGR	MU	NCGR	NCGR	NCGR	UCD	NCGR
**Sequencing**							
Paired end reads	264,112,180	846,241,271	249,382,312	260,534,438	787,524,840	219,992,493	260,634,420
Mate-pairs	71,229,807	57,101,723	75,354,980	78,329,874	54,720,606	63,339,005	82,902,639
***k*-mer analysis**							
Total 31-mers	5.77×10^10^	5.54×10^10^	5.42×10^10^	5.71×10^10^	5.65×10^10^	5.71×10^10^	5.58×10^10^
Haploid 31-mer depth	n/a	24	23	23	24	25	23
Diploid 31-mer depth	50	47	47	47	47	50	47
Genome size estimate^1^	5.77×10^8^	5.83×10^8^	5.82×10^8^	5.71×10^8^	5.94×10^8^	5.71×10^8^	6.00×10^8^
Relative heterozygosity	6	3	5	2	3	4	1

1 The genome size estimates for these the genomes are derived from the paired end sequence using 31-mer histograms as described in methods.

**Figure 1:**
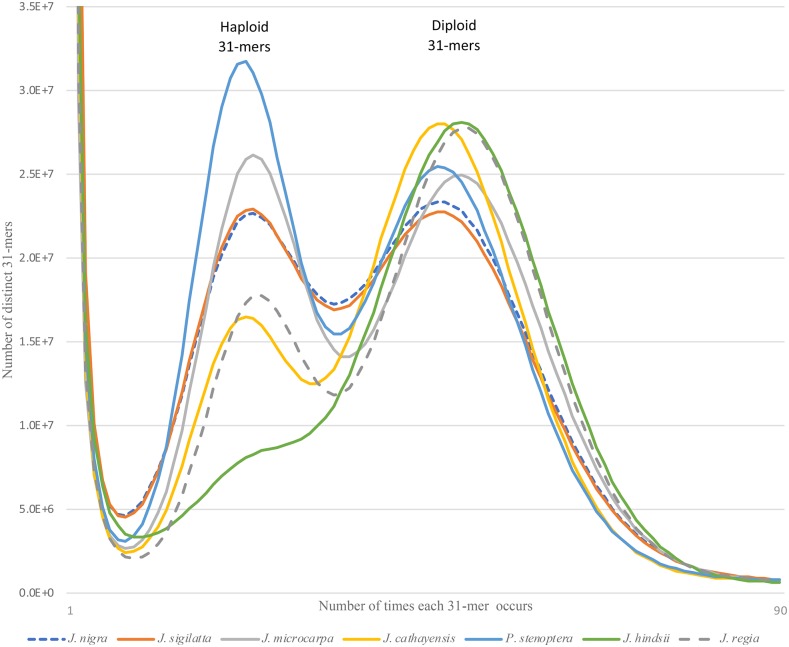
The 31-mer histograms of our paired end sequence data. Each histogram shows a bimodal distribution typical of diploid heterozygous genome. The relative fraction of the distribution under the left (haploid) peak is proportional to the genome heterozygosity. Using the relative proportions of the two peaks the genomes can be ranked by their heterozygosity (Table 1).

Prior to assembly, we characterized the genome using the distribution of all short subsequences of fixed length *k* (*k*-mers) in the unassembled paired end Illumina reads. This was performed using *k*-mer histograms constructed from the paired data using jellyfish (Marçais and Kingsford 2011) with word size (*k*) of 31. The histograms for each genome (Figure 1), display three distinct peaks. The extreme peak at the origin of the depth axis, representing approximately 1% of the distinct 31-mers, are very rare k-mers in the data. These are attributed to sequence errors. The two peaks of interest, together comprising the largest area of each histogram, reflect the bi-modal distribution expected from a heterozygous diploid genome. The area under the right (deeper coverage) “diploid” peak, represent 31-mers shared between the homologous chromosomes. The area under the left (lower coverage) “haploid” peak, represents 31-mers found in only one of the two homologous chromosomes. Among the genomes, the deeper “diploid” peaks occur consistently at twice the depth as the lower coverage “haploid” peaks. However, different genomes exhibited noticeable variation in the relative proportions of the genome found at *haploid* depth, reflecting the variation in the level of heterozygosity among the species. The greater number of differences between chromosomes leads to a larger fraction of haploid-depth 31-mers. Estimates of genome size based on the 31-mer histograms, ranged from a low of 498 Mbp for *J. nigra* to a high of 594 Mbp for *P. stenoptera* (Table 2). The only cytological estimate of genome size for *Juglans* is 606 Mbp for *J. regia* (Horjales 2003). Within the family *Juglandaceae*, the other available C-value estimate is a genome size of 808 Mbp for pecan (*Carya illinoensis*).

**Table 2:** Assembly statistics for our six genomes. The original v1.0 *J. regia* assembly, constructed using similar methods, is included for comparison. As an additional resource and for validation purposes, we also included a v1.5 *J. regia* assembly which incorporates light coverage of PacBio sequences for improved contiguity

Species	Assembly size	Scaffolds	N50 Scaffold
*J. nigra*	640,895,151	232,579	244,921
*J. hindsii*	643,318,433	273,094	470,924
*J. cathayensis*	797,890,490	332,634	145,095
*J. microcarpa*	941,867,385	329,873	135,837
*J. sigillata*	668,759,554	282,224	200,575
*P. stenoptera*	991,966,387	396,056	148,559
*J. regia v1.0*	712,759,961	186,636	241,714
*J. regia v1.5*	651,682,552	4,402	639,948

### Genome assemblies

The draft genome assemblies for the species sequenced and assembled for this paper are characterized in Table 2. The statistics are compared to the original *Juglans regia* v1.0 assembly. It is notable that variance in assembly size is clearly higher than the variance in estimated genome sizes (Table 1). In particular, there are two instances where the genome was much larger than expected: *Juglans microcarpa* and *Pterocarya stenoptera*. These two species are also outliers for a number of additional measures which we describe below. An unannotated v1.5 *J. regia* assembly, incorporating light coverage of long PacBio sequences for improved contiguity (N50 of 639 kbp *vs.* 242 kbp), is included as an additional resource and to validate inferences made from the v1.0 *J. regia* assembly.

### Core gene annotation

Both the Benchmarking Universal Single-Copy Orthologs (BUSCO) (Simão *et al.* 2015) and the Core Eukaryotic Genes Mapping Approach (CEGMA) (Parra *et al.* 2007) were used to estimate the completeness of the new assemblies. For all of the species, the final assemblies compared favorably with the original *J. regia* reference genome (Martínez-García *et al.* 2016) (Table 3). The CEGMA results are given for 248 eukayotic genes. The most general metric for assembly completeness, the percentage of CEGs annotated as complete or partial ranges from 93.55% (*J. nigra*) to 96.37% (*J. sigillata*), with *J. regia* falling in the middle of that range at 94.76%. The BUSCO analysis is aimed at a more targeted phylogenetic range and includes more genes. The BUSCO results are given for 1440 single copy Embryophyte genes. A comparable measure of completeness, including fragmented annotations, has a slightly narrower range from 94.24% (*J. microcarpa*) to 96.18% (*J. regia*). BUSCO additionally estimates the fraction of single copy orthologs that appear duplicated in the target genome. The genomes of *J. microcarpa* and *P. stenoptera* also stood out as having high values for this statistic, more than twice the average of the other genomes.

**Table 3:** CEGMA Core gene results for the genome assemblies of all six *Juglans* species and the outgroup *P. stenoptera*. CEGMA: Complete and Partial record the number and fraction of all 248 ultra-conserved CEGs present in the assembly as a complete or partial annotation respectively. Partial annotations use a more liberal cutoff that includes all complete annotations. BUSCO: The number and percentage of 1440 single copy Embryophyte genes present in the assembly. These results are further broken down into single-copy and duplicated genes. *Summary results for v1.0 and v1.5 assemblies were the same for both analyses

	*J. cathayensis*	*J. nigra*	J. regia*	*J. hindsii*	*J. sigillata*	*J. microcarpa*	*P. stenoptera*
CEGMA							
Complete	207	201	206	203	201	201	205
>%	83.47	81.05	83.06	81.85	81.05	81.05	82.66
Partial	235	232	235	239	238	238	234
%	94.76	93.55	94.76	96.37	95.97	95.97	94.35
BUSCO							
Complete	1330	1346	1370	1357	1343	1320	1323
%	92%	93%	95%	94%	93%	92%	92%
Single-copy	1005	1198	1071	1187	1185	780	743
%	70%	83%	74%	82%	82%	54%	52%
Duplicated	325	148	299	170	158	540	580
%	23%	10%	21%	12%	11%	38%	40%
Fragmented	32	26	14	28	28	37	41
%	2%	2%	1%	2%	2%	3%	3%
Missing	78	68	56	55	69	83	76
%	5%	5%	4%	4%	5%	6%	5%

### Pairwise Genome Alignments and Genome-wide Phylogenies

Pairwise alignments were constructed as a resource to evaluate micro-synteny and sequence conservation (divergence) between pairs of species. The alignment methodology used was asymmetric and the alignments between a pair of genomes differed depending upon which genome was used as the query and which was the reference. The filtered alignments consisted of a tiling path of aligned segments with respect to the reference genome. The aligned coverage and corresponding divergence estimates for each possible query reference assignment of seven genomes, presented in Table 4, supports the recognized section level classifications of the species (Table 4). Coverage was typically quite high, 80–90% of sites (dark gray), for pairs of *Juglans* species within the same section, and dropped to as low as 60% (light gray) between *Juglans* species pairs in different sections. Alignments between *Juglans* species and the *Pterocarya stenoptera* outgroup had the lowest coverage. Pairwise divergence estimates varied for the most part inversely with alignment coverage. However, this relationship breaks down for the distant comparisons involving *P. stenoptera* that have the largest fractions of unaligned bases.

**Table 4:** Pairwise genome alignment statistics (top) The percent coverage is calculated for each ordered pair as the percentage of the reference genome covered by the aligned query genome. (bottom) Divergence is calculated for each ordered pair of aligned query to reference genomes. For both metrics, the highest values belonged to pairs of genomes within the same *Juglans* section

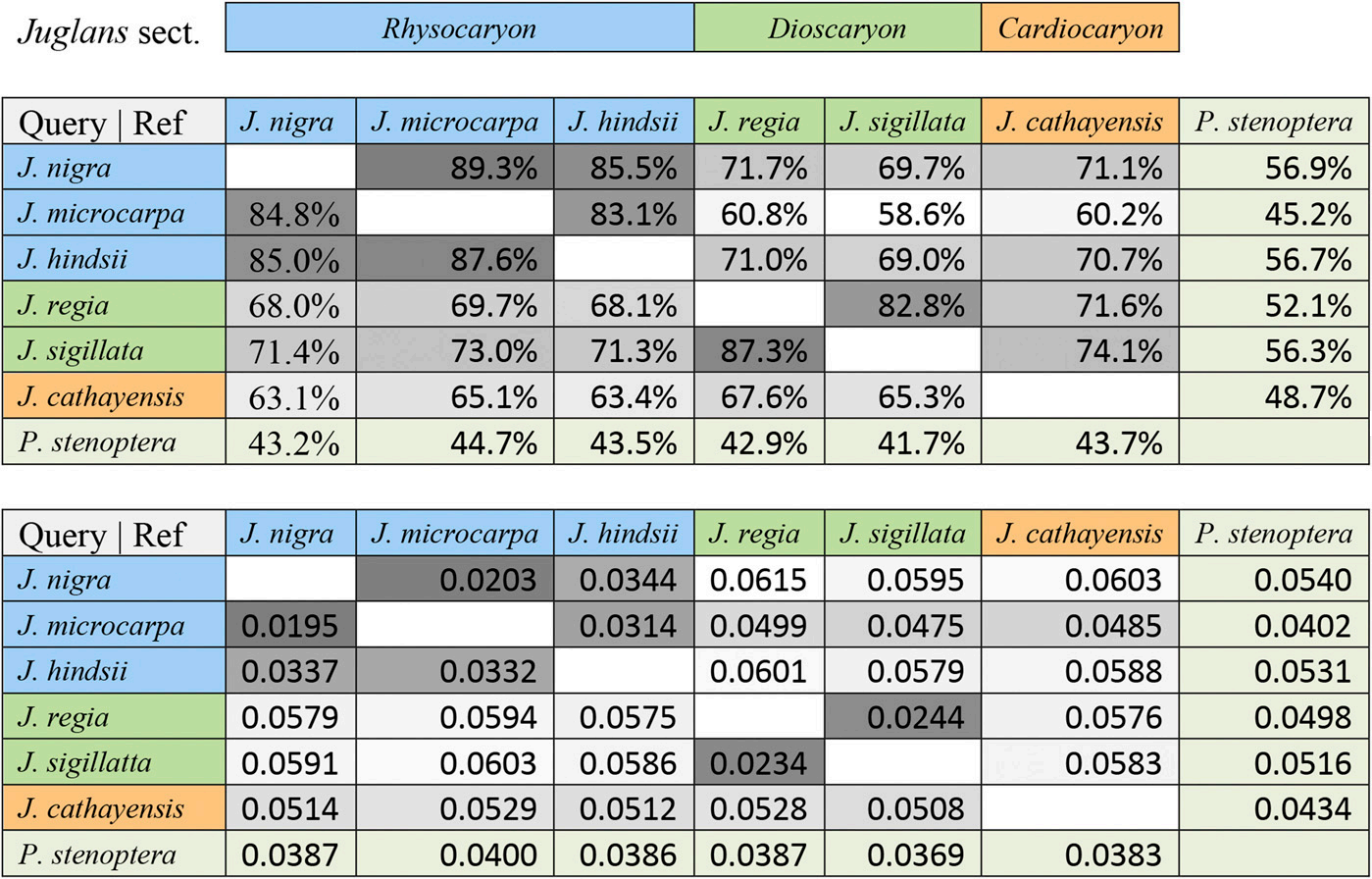

**Figure 2:**
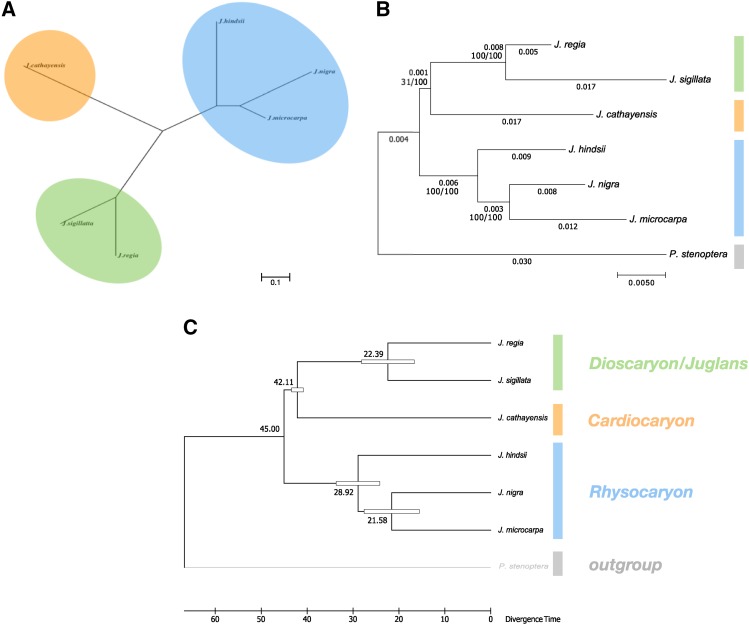
Genome wide phylogenetic trees. (a) An unrooted neighbor joining tree reconstructed from genome wide pairwise divergence estimates. The tree is drawn to scale with the bar representing 0.1 nucleotide substitutions per site. (b) Rooted maximum likelihood trees constructed from the curated nucleotide alignments of single copy BUSCO orthologs appearing in all seven genomes. The scale bar represents 0.005 nucleotide substitutions per site. Nucleotide distances and the number of bootstrap replicates supporting the split are noted on each edge. (c) *Juglans* chronogram calibrated from (b) estimating section level divergence times (MYA).

We estimated two classes of genome-wide phylogenetic trees from our data. The most broadly informed tree uses pairwise Jukes-Cantor divergence estimates obtained from the genome alignments given in Table 4. These divergence estimates were calculated from the concatenated forward and reciprocal alignments between pairs of species. We excluded P. stenoptera from this tree because of obvious ascertainment bias; the aligned coverage was much more restricted to the most conserved regions of the genome. The resulting neighbor joining tree (Figure 2a) can be compared to two additional trees constructed using only orthologous single copy genes and incorporating the outgroup. These trees were estimated using the set of BUSCO orthologs present as a single copy in all seven genomes from which a good multiple alignment could be obtained. Concatenated multiple alignments of 244 gene sequences were used to reconstruct the maximum likelihood phylogeny in Figure 2b. When restricted to these highly conserved genes, the total divergence in the genus Juglans was notably much less than captured in the unrooted tree constructed from the pairwise genome alignments. Genome wide phylogenetic trees were consistent with the accepted section level classifications of Dioscaryon, Rhysocaryon, and Cardiocaryon. A chronogram was constructed (Figure 2c) to estimate section level divergence times and compare them to previous results Dong *et al.* (2017). For comparison, the calibration point of 45Mya for the ancestor of the Juglans (Dong *et al.* 2017) was used.

### Genomic diversity in J. regia, J. hindsii, J. nigra and J. microcarpa

Table 5 shows the numbers of SNPs identified in *J. regia*, *J. hindsii*, *J. nigra* and *J. microcarpa* and corresponding estimates of the nucleotide diversity. These can be compared to the relative heterozygosity rankings in Table 1. As inferred from the 31-mer analysis (above) *J*. *nigra and J. microcarpa* harbor more SNPs and have higher estimates of expected heterozygosity that do *J. regia* and *J. hindsii*. The 31-mer analysis indicates our *J. microcarpa* genome is more heterozygous than *J. nigra*. The lower nucleotide diversity estimate for *J. microcarpa* may be due to the lower re-sequencing coverage obtained compared to *J. nigra*. As demonstrated below these SNPs are not only resources for genotyping in breeding efforts, they can be critical resources in the identification of impact of natural and artificial selection on and around specific genes associated with traits of interest. It is also important to recognize that these SNP resource capture the majority of the diversity in the species and in particular that in ongoing breeding programs.

**Table 5:** The count of single nucleotide polymorphisms and a corresponding estimate of nucleotide diversity from re-sequenced population samples from four *Juglans* species. The individual accessions are described in Supplementary Table S2

Species	Number of individuals	Re-sequenced depth	Filtered single nucleotide polymorphisms	Nucleotide diversity π
*Juglans hindsii*	10	90.8X	942,379	**π** = 0.0016
*Juglans microcarpa*	12	87.2X	4,427,957	**π** = 0.0089
*Juglans nigra*	13	1525X	11,003,383	**π** = 0.0096
*Juglans regia*	27	1620X	9,619,940	**π** = 0.0056

### The evolutionary history of the polyphenol oxidase (PPO) genes

**Figure 3:**
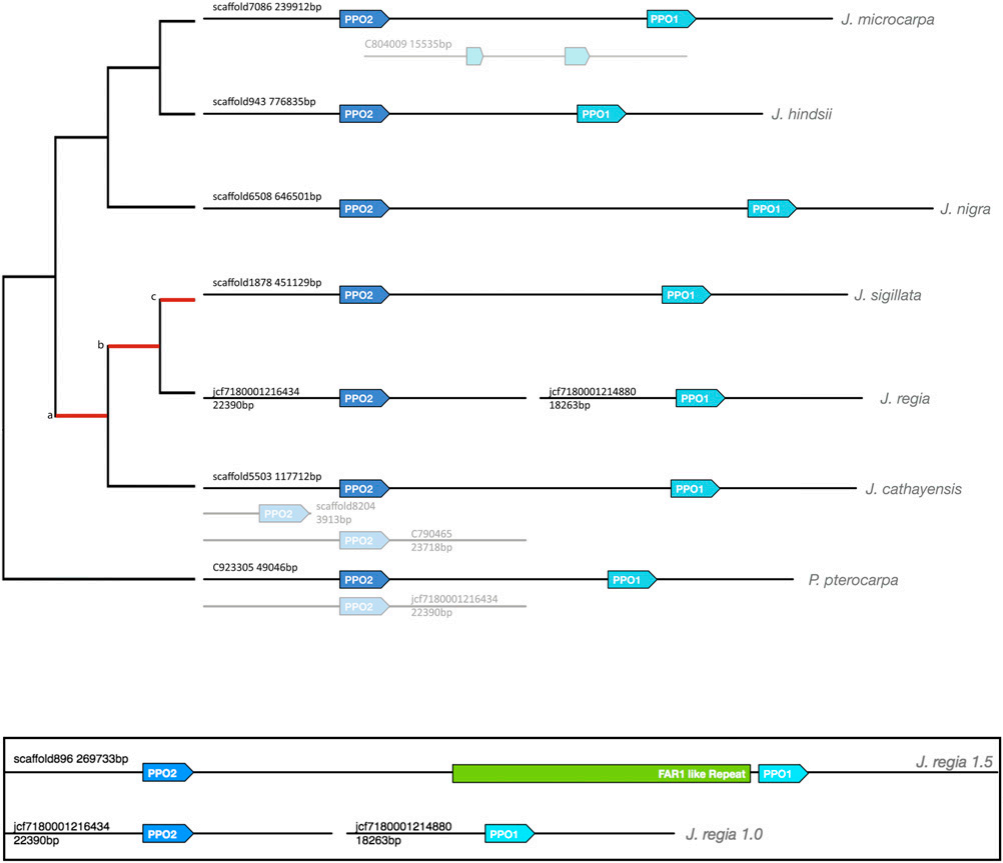
Orthologs, paralogs, and alleles of *PPO1* and *PPO2* in the six *Juglans* species and the outgroup *P. stenoptera*. Figure illustrates the location, order, and orientation of the PPO gene family in each assembly. Copies identified as haploid alleles are gray. A allelic copy of *PPO1* interrupted by an insertion was also noted in *J. microcarpa*. Lineages with positive *K_a_/K_s_* are marked in red on the dendogram to the right. [a,b] *PPO1 K_a_/K_s_* 0.006/0.002 *PPO2 K_a_/K_s_* 0.001/0 [b,c] *PPO1 K_a_/K_s_* 0.03/0.01 [c,] *PPO1 K_a_/K_s_* 0.002/0. **Inset**: Comparing *J.regia* v1.5 (top) and v1.0 (bottom). In *J.regia* v1.5 the two genes are tandem and the contiguous interval between reveals a novel repetitive sequence with homology to FAR1 and the potential cause of the original assembly issue.

In Martínez-García *et al.* (2016) the reference genome sequence of ‘Chandler’ was used to first identify and characterize *two* distinct polyphenol oxidase (PPO) genes (*PPO1* and *PPO2*) in *Juglans regia*. The genes were on separate genomic scaffolds, neither linked to a chromosome. Here we use comparative genomics resources to characterize PPO genes in six *Juglans* species and the outgroup *Pterocarya stenoptera*. In all seven species, we observe a copy of both *PPO1* and *PPO2* in close proximity, in the same relative orientation, on the same assembly scaffold (Figure 3). In *J. cathayensis* and *P. stenoptera* we found extra copies of *PPO2*. For these extra copies, we used k-mer depth to determine that the extra copies were due to assembly artifacts and represent alleles of the heterozygous gene (Table S5). In *J. microcarpa* we found an apparent allelic copy of *PPO1* interrupted by an insertion.

**Figure 4:**
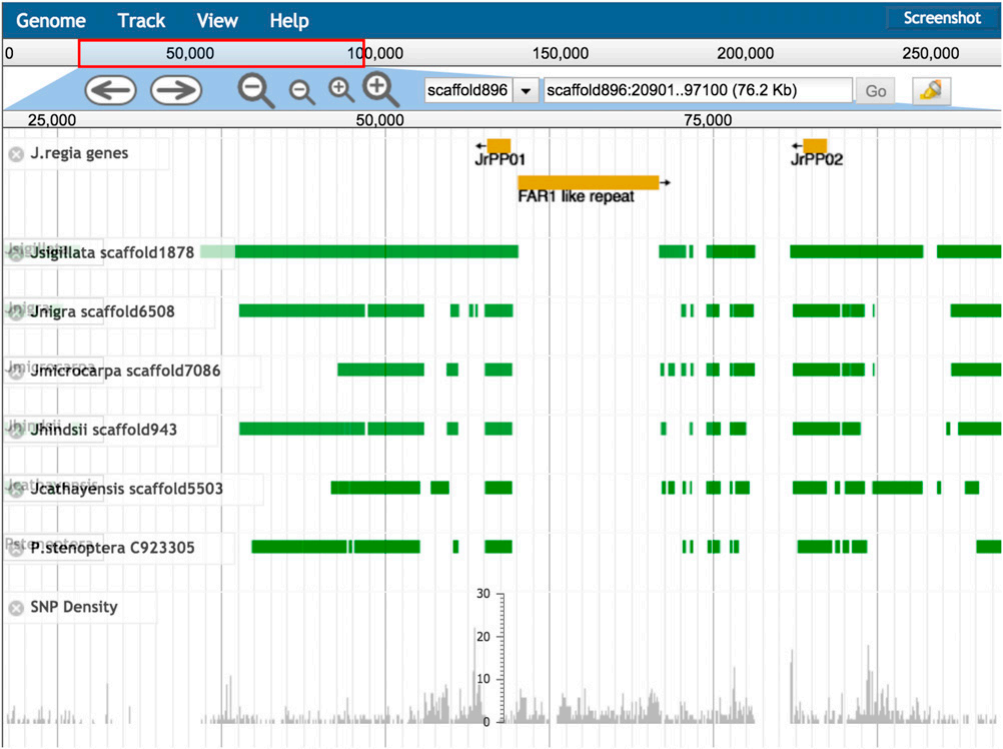
Desktop genome browser sessions using JBrowse. The PPO1 and PPO2 region of scaffold896 in *J. regia* v1.5. The gene regions for PPO1 and PPO2 are aligned to the same scaffold in assemblies as divergent as the outgroup *P. stenoptera*. An apparent excess divergence in *J. regia* coincides with a lineage specific insertion of a 10kbp FAR1 domain containing repeat. At this scale only SNP density is visible. Zooming in would reveal the 8 sites overlapping PPO1 and the 20 sites overlapping PPO2.

To confirm our findings with micro-synteny, we examined the directed pairwise genome alignments for reciprocity and found that the genomic region containing *PPO1* and *PPO2* was conserved and co-linearly aligned (syntenic) across the two tandem genes (Figure 4). Taken together the results of protein homology, micro-synteny, and k-mer depth, suggest that single functional copies *PPO1* and *PPO2* genes are in fact tandem in all *Juglans* species examined, consistent with an ancestral gene duplication.

PPO1 and 2 are classical type 3 Cu-binding proteins that catalyze the oxidation of mono and ortho-di-phenolic substrates. As noted in Martínez-García *et al*. 2016 and confirmed here, they are differentially expressed in different *J. regia* tissue types with *PPO1* being the most abundantly expressed of the two genes in terms of expression in a wider range of tissues (Supplementary table 6). Not surprisingly, *PPO1* was the first characterized and shown to be able to convert both monophenolic substrates like tyrosine and diphenolic substrates like DOPA into melanin pigments (Escobar *et al.* 2008). *PPO1* is upregulated by jasmonic acid (Escobar *et al.* 2008) and in response to walnut blight infection (Khodadadi *et al.* 2016). The higher expression *PPO1* does not appear to be limited to *Juglans regia* (Supplementary table 7). Structural alignment of primary sequence of *PPO1* and *PPO2* show correspondence between all relevant structural features. Both proteins have two transit peptides indicating chloroplast localization. Superposition of *PPO2* modeled with the recently solved structure of *PPO1* reveal conservation of the active site cavity indicating that *PPO2* would also display mono and diphenolic activity (Supplementary figure 2). However, the replacement of a few residues in the mouth of the active site results in a change in the electrostatics of the surface that could result an altered range of substrate specificity. Using an outgroup we can infer these consequential changes likely happened on the lineages leading to *PPO1* (Supplementary figure 3).

The pairwise genome alignments also identified a 10kbp insertion in the *J. regia* 1.5 assembly that was not present in the other genomes. We annotated the inserted sequence as a repetitive element, noting that the complete sequence is present as insertions in both the *J. regia* and *J. sigillata* genomes at multiple unrelated loci. The three loci in *J. regia* and one in *J. sigillata* all appeared to be lineage specific locations. Those in *J. regia* are apparently homozygous based on examination of the raw Illumina reads. No complete elements were detected in other *Juglans* genomes. The sequence of the inserted repeat adjacent to *PPO1* contains a truncated open reading frame annotated with a DNA-binding domain FAR1 (PF03101). The FAR1 domain functions as a transcription factor (Hudson *et al.* 2003) in other contexts. While the potential functional impact of the inserted repeat in requires further empirical study, the sequence is a likely cause for the break in the v1.0 assembly between the PPO genes.

An outward taxonomic search for the ancestor of the gene duplication led us to a *PPO2* ortholog in the genome of Valley Oak (*Quercus lobata)* (Sork *et al.* 2016). The complete *Q. lobata* genome contained no corresponding ortholog of *PPO1*. A phylogenetic analysis of these PPO genes was undertaken and a maximum likelihood gene tree was constructed from which divergence values were estimated. The gene tree shows that subsequent to the duplication event, the *PPO1* subgroup diverged more rapidly from the common ancestor than did the *PPO2* subgroup (Supplementary figure 1). Tajima’s relative rate test (Tajima 1993) comparing *JrPPO1* to *JrPPO2* using *QlPPO* as an outgroup yielded a significant excess of differences along the *JrPPO1* lineage (*P* < 0.0003). The most pronounced difference in rates occurs on the lineage immediately following to the common ancestor of section *Juglans* (1) and (2), when nine times as many changes (46 *vs.* 5; *P* < 1e-6) occurred to the *PPO1* gene compared to *PPO2*. A recent acceleration in the nucleotide divergence rate in Juglans *PPO1* is inferred when these rates of nucleotide change are compared to the lineages derived immediately from the PPO common ancestor. Immediately after duplication, only 1.27 times as many changes (*P* < 0.003) happened on the *PPO1* lineage compared to *PPO2*. High ratios of nonsynonymous to synonymous substitution were observed in *PPO1* on the lineages leading to *J. regia* and to *J. sigillata* (Section *Juglans*), especially the lineage to their common ancestor where *K_a_* = 0.03 and *K_s_* = 0.01. High ratios of nonsynonymous to synonymous substitutions (*K_a_/K_s_*) indicate selection.

The relatively low level of polymorphism in *J. regia* at the *PPO1* locus relative to divergence compared and to the same quantities measured in and around *PPO2* are consistent with the ‘hitchhiking effect’ (Maynard Smith and Haigh 1974) of recurrent directional selection and suggest that the impact of domestication continues to the present. In the resequencing data from 27 *J. regia* samples (Table 5; Table S2) we observed reduced polymorphism at the PPO1 locus: 8 segregating sites compared to 20 at *PPO2*. Using an HKA-like test for selection (Hudson *et al.* 1987), the reduction in polymorphism was determined to be significant (*P* < 3x10^−6^) when polymorphism is compared to the estimated divergence in section *Juglans*: 53 nucleotide changes for *PPO1* and 13 nucleotide changes for *PPO2*.

Addressing the question of when the ancestral PPO duplication occurred informs which additional taxa may contain descendants of the duplicated genes. Subject to the caveats associated with chronogram analyses, lineage lengths on the PPO gene tree (Figure S1) indicate that the ancestral PPO gene duplication occurred near the basal split of the *Juglandaceae* and *Fagaceae* families. Estimates based on fossil records place the ancestor of *Juglandaceae* at 71-96 MYA (Xiang *et al.* 2014), which is an upper estimate for the age of the gene duplication. A lower estimate for the age of the gene duplication would be the divergence of *Pterocarya* and *Juglans* during the late Paleocene/Lower Eocene approximately 54MYA (Manchester 1987). Consistent with these findings, using the same methodology, single copy orthologs of *JrPPO1* and *JrPPO2* were annotated in an unpublished assembly of Pecan (*Carya illinoinensis* var. Pawnee)) (Jenkins *et al.* 2015) obtained from the HudsonAlpha Institute (hudsonalpha.org). This additional observation shows that the duplication also predated the split of *Carya* and *Juglans* during the *Paleocene* 60MYA (Manchester 1987).

## Discussion

### Quality and Completeness of the Draft Genomes

For the six new assemblies, gene space completeness, as estimated by both BUSCO and CEGMA, was comparable to the original *J. regia* V1.0 assembly. This result is consistent with the similarity in methodology used to obtain the assemblies. Heterozygous diploid genomes are a challenge for genome assemblers. The observation that the inflated assemblies were positively associated with the genomes with higher estimated heterozygosity fits a hypothesis that heterozygosity is the underlying cause for assembly inflation. To varying degrees, these genome assemblies consist of regions that are a diploid consensus and regions that are haploid alleles. The unintended haploid regions are a consequence of divergent haplotypes that were neither collapsed by the assembler nor filtered downstream. This haploid allele inflation is most notable in the assemblies of *P. stenoptera* and *J. microcarpa*. These two species had the highest level of heterozygosity in the unassembled reads (Table 1) and their assemblies had the highest levels of inflation over their estimated genome size (Table 2). These two genomes also had the highest levels of duplicated, single copy BUSCO orthologs (Table 3). We presume that additional allelic copies of the single copy genes are present in these genomes.

### New Juglans genomes are useful for gene-oriented analysis

The potential for gene-oriented analysis of the genomic variation in these *Juglans* reference sequences, their pair-wise alignments, and in the polymorphism data, is evident in our investigation of the PPO genes:

1. The number, spacing and orientation of *PPO1* and *PPO2* is conserved in the *Juglans* genomes and *Pterocarya stenoptera*.
2. In a more distant lineage, *Quercus*, a single PPO gene was found in the genome.
3. The more rapid divergence, compared to *PPO2*, and the lack of an ortholog in *Quercus* is consistent with a “derived” *PPO1*.
4. As has been reported for other seed crops, excess *K_a_/K_s_* in the divergence of PPO1 on the sect. *Dioscaryon* lineage supports the view that domestication often involves selection on traits determined by PPO activity, *e.g.*, biosynthesis of phenols, color, pest resistance, etc.

### Accessibility of the new genomes

To analyze the PPO genes in *Juglans* we applied a variety of bioinformatics tools to the new *Juglans* genomics resources. But the accessibility and utility of the different software varies greatly. Many software tools cannot be usefully applied on a genomic scale, nor can their results be readily inspected. To demonstrate how the genomic resources described in this paper can be browsed quickly to gain the basic comparative, gene-oriented analyses we developed a simple method to load these data into the widely used and well-supported JBrowse software (Skinner *et al.* 2009) [Figure 4, which show the *PPO1* and *PPO2* region of *J. regia v1.5* (scaffold 896)]. Below the annotated *PPO1* and *PPO2* genes, are the nucmer pair-wise alignments of the *PPO1* gene region of the other species to the *J. regia* scaffold showing the decreasing levels of alignment coverage to the other species with increased divergence. The illustration highlights the synteny of the two PPO genes within *Juglans* and with *Pterocarya*. It further highlights the location and size of the *FAR1*-like repeat, an approximately 10kbp insertion in *J. regia* that is not present in the other genomes. At the bottom of the figure SNPs from the *J. regia* vcf file are displayed in gray as a density histogram. We imagined a scenario in which a researcher wished to identify, *e.g.*, using a BLAST search, a scaffold in one or more *Juglans* species that may have homology to a sequence of a known gene that could be from any species. First an identified scaffold can be loaded into JBrowse. Then all alignments of the other *Juglans* species (including *P*. *stenoptera*) can be loaded. Other resources in a large number of formats (*e.g.*, gff, bam, vcf, bed) can be loaded in the coordinate system of the target scaffold, including genotyping data from arrays or GBS. The entire resource of reference sequences, pair-wise alignments and SNP gff files can easily fit on a personal computer, allowing the exploration structures and divergence of all the *Juglans* genomes and their within species polymorphism. A user can also add annotation as discoveries are made. High quality gene annotation, when it becomes available will further enhance these resources for *Juglans* researchers and breeders.

### Alignments & SNPs

The genome alignments included here are a resource for divergence annotation and for identifying micro-synteny. We used the divergence estimates to construct genome wide phylogenies from the data. We used the micro-synteny annotation to validate a tandem duplication hypothesis for the history of the PPO gene family.

While we did not construct a multiple alignment, for many analyses, pairwise alignments will be sufficient to polarize analyses of polymorphism or obtain site specific divergence values. Once these genomes are fully annotated, determining which genes harbor variants likely to have phenotypic effect and exhibit patterns consistent with strong selection will be of great interest, particularly for those lineages undergoing domestication.

For *J. regia*, *J. nigra*, *J. microcarpa*, *and J. hindsii*, we computed a set of filtered genome wide SNPs and quantified the variation within each species. We observed high levels of nucleotide diversity for *J. microcarpa* and *J. nigra* and low levels in, *J. regia* and *J. hindsii* consistent with initial estimates from 31-mer histograms. The lowest level of heterozygosity was observed in *J. hindsii*. This is consistent with the limited natural range and small population of the endemic species. The restricted demographic history for *J. hindsii* was first proposed by McGranahan *et al.* (1988). The low heterozygosity observed here confirms previous estimates based on RFLP markers (Fjellstrom and Parfitt 1994) and SSRs (Ross-Davis and Woeste 1998).

The SNPs included here are rich resource of potential markers of interest to breeding programs. In the design of genotyping platforms to greatly augment scion and rootstock breeding, SNPs can be selected based on quality of the SNP call, sequencing depth, allele frequency, sample size, depth, and physical linkage. The relatively complete, unphased genotypes creates a foundation for developing, implementation and validation of GBS tools.
